# Comparative Analysis of the Complete Chloroplast Genomes of Four *Aconitum* Medicinal Species

**DOI:** 10.3390/molecules23051015

**Published:** 2018-04-26

**Authors:** Jing Meng, Xuepei Li, Hongtao Li, Junbo Yang, Hong Wang, Jun He

**Affiliations:** 1 College of Horticulture and Landscape, Yunnan Agricultural University, Kunming 650201, China; lixuepeix@163.com; 2 Germplasm Bank of Wild Species, Kunming Institute of Botany, Chinese Academy of Sciences, Kunming 650201, China; lihongtao@mail.kib.ac.cn (H.L.); jbyang@mail.kib.ac.cn (J.Y.); 3 Key Laboratory for Plant Diversity and Biogeography of East Asia, Kunming Institute of Botany, Chinese Academy of Sciences, Kunming 650201, China; wanghong@mail.kib.ac.cn

**Keywords:** ranunculaceae, ‘Dula’, comparative genomics, herbal medicine, phylogenetic analysis

## Abstract

*Aconitum* (Ranunculaceae) consists of approximately 400 species distributed in the temperate regions of the northern hemisphere. Many species are well-known herbs, mainly used for analgesia and anti-inflammatory purposes. This genus is well represented in China and has gained widespread attention for its toxicity and detoxification properties. In southwestern China, several *Aconitum* species, called ‘Dula’ in the Yi Nationality, were often used to control the poisonous effects of other *Aconitum* plants. In this study, the complete chloroplast (cp) genomes of these species were determined for the first time through Illumina paired-end sequencing. Our results indicate that their cp genomes ranged from 151,214 bp (*A. episcopale*) to 155,769 bp (*A. delavayi*) in length. A total of 111–112 unique genes were identified, including 85 protein-coding genes, 36–37 tRNA genes and eight ribosomal RNA genes (rRNA). We also analyzed codon usage, IR expansion or contraction and simple sequence repeats in the cp genomes. Eight variable regions were identified and these may potentially be useful as specific DNA barcodes for species identification of *Aconitum*. Phylogenetic analysis revealed that all five studied species formed a new clade and were resolved with 100% bootstrap support. This study will provide genomic resources and potential plastid markers for DNA barcoding, further taxonomy and germplasm exploration of *Aconitum*.

## 1. Introduction

Chloroplasts are an important semiautonomous organelle in plants, providing essential energy [1]. The chloroplast genome structure, gene content and gene order is often better conserved than the nucleus and the mitochondria genome. It contains about 130 genes, with a typical circular quadripartite structure comprising two identical copies of inverted repeats (IRs), separated by a large single-copy region (LSC) and a small single-copy region (SSC). The chloroplast genome sizes of almost all land plants range between 120–160 kb in length [2,3]. Generally, coding regions in the chloroplast genome show lower polymorphism than non-coding regions [4]. With the rapid development of next generation sequencing (NGS) technologies, an increasing number of studies have focused on plant chloroplast genomes. Sequencing and phylogenetic analysis on the complete cp genome is a highly efficient and relatively low-cost way for improving intrageneric classification and population analysis. Recently, comparative analysis of the complete chloroplast (cp) genomes of several closely related species has provided promising results for the study of phylogeny, species identification and evolution [3,5,6,7,8,9]. 

*Aconitum* (Ranunculaceae) is a perennial or pseudoannual genus with an erect or twining stem and blue, purple or yellow flowers. It is comprised of about 400 species distributed in the temperate regions of the northern hemisphere and there are 211 species in China [10]. The genus is one of the most important medicinal and poisonous plants in the world [10]. *Aconitum* species have been noted for their toxicity from as early as mid-7 BC in Guoyu [11]. At present, many species are well-known herbs and mainly used for analgesia and anti-inflammatory purposes [12]. To date, the cp genomes of 17 *Aconitum* species have been reported [13,14].

In southwestern China, some *Aconitum* species, which are called ‘Dula,’ are often used to control the poisonous effects of other *Aconitum* plants; they are *A. episcopale*, *A. vilmorinianum*, *A. contortum* and *A. delavayi* [15,16,17,18,19]. The first two species are twining, belonging to *Aconitum* subgen. *Aconitum* ser. *Volubilia*; the last two species are erect and a member of ser. *Stylosa* and ser. *Ambigua* respectively [20]. The major medicinal and toxic compounds aconitine, hypaconitine and mesaconitine are not contained in these species. However, the use of morphological and molecular markers for the identification of *Aconitum* species is controversial or limited due to unmanageable phenotypic characteristics or morphological similarity among species [21,22,23,24]. The coexistence of toxic species and species with antidote properties in *Aconitum* may result in misuse during practical application. Therefore, providing more genomic information is imperative for the understanding of these species and the safe and effective utilization of ‘Dula.’ 

In this study, we used an Illumina Miseq Platform to assemble the cp genomes of five herbal plants in *Aconitum*, four ‘Dula’ (*A. episcopale*, *A. contortum*, *A. vilmorinianum*, *A. delavayi*) and one, *A. hemsleyanum* (ser. *Volubilia*), which is also twining and morphologically similar to *A. vilmorinianum* and clustered with ‘Dula’ in previous analyses [23,24,25]. The aims of our study were: (1) to deepen understanding of the structural patterns of *Aconitum* cp genomes; (2) to provide knowledge for species identification of ‘Dula’; (3) and to reconstruct phylogenetic relationships among the *Aconitum* species using the cp genome sequences. 

## 2. Results and Discussion

### 2.1. Chloroplast Genome Features

**Figure 1:**
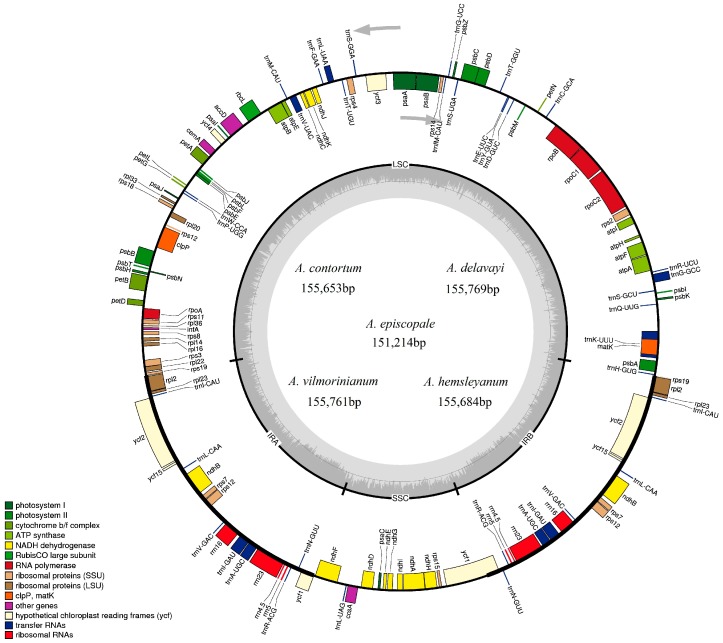
Chloroplast genome map of five *Aconitum* species. Genes lying outside the circle are transcribed in the counter clockwise direction, while those inside are transcribed in the clockwise direction. The colored bars indicate different functional groups. The darker gray area in the inner circle denotes GC content while the lighter gray corresponds to the AT content of the genome. LSC: large single copy, SSC: Small single copy, IR: inverted repeat.

We obtained cleaned reads of 8,783,602 bp to 24,899,740 bp from the five *Aconitum* species, using the Illumina Miseq platform. Out of 1161–2540 de-novo assembled contigs, only 3–4 contigs were used in the final cp genome assemblies (Table 1). The five *Aconitum* cp genomes ranged from 151,214 bp (*A. episcopale*) to 155,769 bp (*A. delavayi*) in length and 56.4× to 159.9× coverage. They had a typical quadripartite structure that was similar to the majority of cp genomes of land plants, consisting of a pair of IRs (26,209 bp–26,240 bp), separated by the LSC (83,182 bp–86,394 bp) and SSC (of 15,598 bp–16,949 bp) regions (Table 1 and Figure 1). Compared with other *Aconitum* species, we discovered that the size of the cp genome in *A. episcopale* is the smallest [13,14]. These five plastomes are highly conserved in gene content, gene order and intron number. The cp genomes of *A. vilmorinianum*, *A. delavayi*, *A. hemsleyanum* and *A. contortum* had the same GC content of 38.1%, *A. episcopale* showed a subtle difference (38.3%) compared with the others. The GC contents of the LSC and SSC regions of the five species were lower than that of the IR regions due to the reduction of AT nucleotides in the four duplicate rRNA genes [6,26]. 

The cp genomes of four species contained 112 unique genes, including 78 protein-coding genes (PCGs), 30 transfer RNA genes (tRNA) and four ribosomal RNA genes (rRNA). *A. episcopale* did not contain the *trnG-UCC* tRNA gene. The LSC region contained 60 PCGs and 21–22 tRNA genes, while the SSC region contained eleven PCGs and one tRNA gene. Seven PCGs (*rpl2*, *rpl23*, *ycf2*, *ycf15*, *ndhB*, *rps7* and *rps12*), seven tRNA (*trnI-CAU*, *trnL-CAA*, *trnV-GAC*, *trnI-GAU*, *trnA-UGC*, *trnR-ACG* and *trnN-GUU*) and all four rRNA (*rrna16*, *rrna23*, *rrna4.5* and *rrna5*) genes were duplicated and all were located in IR regions (Table 1 and Table 2 and Figure 1). Compared with *A. sinomontanum*, *A. barbatum* var. *puberulum* and *A. barbatum* var. *hispidum*, the *rps16* gene was missing in these five species [13,26]. Two pseudogenes *ψrps19* and *ψycf1* were found in *A. contortum*, *A. episcopale*, *A. vilmorinianum* and *A. hemsleyanum*, while only one pseudogene *ψycf1* was found in *A. delavayi*. 

Introns are non-coding fragments of genes that are under less functional constrains and thus accumulated mutations more rapidly. However, introns can have an integral in regulating gene expression [27]. In four *Aconitum* species, with the exception of *A. episcopale*, 15 intron-containing genes were investigated: 12 genes (*atpF*, *rpoC1*, *ndhB*, *petB*, *rpl2*, *ndhA*, *trnA-UGC*, *trnI-GAU*, *trnK-UUU*, *trnL-UAA*, *trnG-GCC* and *trnV-UAC*) had only one intron, while three genes (*clpP*, *ycf3* and *rps12*) had two introns. In *A. episcopale*, there are also 15 intron-containing genes; the *petB* gene does not contain an intron, the other intron-containing genes are similar to the other four *Aconitum* species (Table S1). The *rps12* gene was a trans-spliced gene with 5’ end located in the LSC region and the duplicated 3’ end in IR regions, which was similar to other land plants [6,9]. The *trnK-UUU* gene had the largest intron and ranged from 2236 bp–2538 bp. It also contained the *matK* gene.

### 2.2. Codon Usage

**Figure 2:**
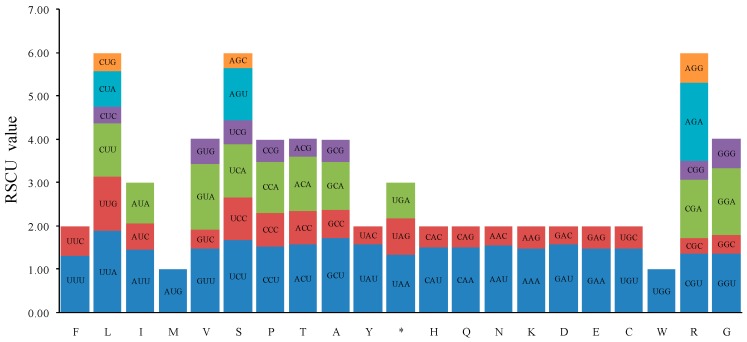
Codon content for the 20 amino acids and stop codons in 85 protein-coding genes in the five *Aconitum* species chloroplast genomes. RSCU: relative synonymous codon usage; F: phenylalanine; L: leucine; I: isoleucine; M: methionine; V: valine; S: serine; P: proline; T: threonine; A: alanine; Y: tyrosine; *: stop; H: histidine; Q: glutamine; N: asparagine; K: lysine; D: aspartic acid; E: glutamic; C: cysteine; W: tryptophan; R: arginine; G: glycine.

We further analyzed the codon usage frequency and relative synonymous codon usage (RSCU) based on sequences of 85 PCGs in the five *Aconitum* species cp genomes. Among these, leucine was the most abundant amino acid, with 2325 (10.32%), 2316 (10.32%), 1852 (8.77%), 2320 (10.32%) and 2324 (10.33%) of codons in *A. vilmorinianum*, *A. delavayi*, *A. episcopale*, *A. hemsleyanum* and *A. contortum*, respectively, while cysteine was the least abundant amino acid, with 256 (1.14%), 256 (1.14%), 382 (1.81%), 256 (1.14%) and 256 (1.14%) codons in each species, respectively (Figure 2 and Table S2). Codon usage was biased towards A and T at the third codon position in the five species, which agrees with previous reports for the angiosperm chloroplast genome [28,29,30]. Furthermore, the usage of start codons AUG and UGG, encoding methionine and tryptophan, had no bias (RSCU = 1) (Table S2).

### 2.3. Repeat and SSR Analyses

**Figure 3:**
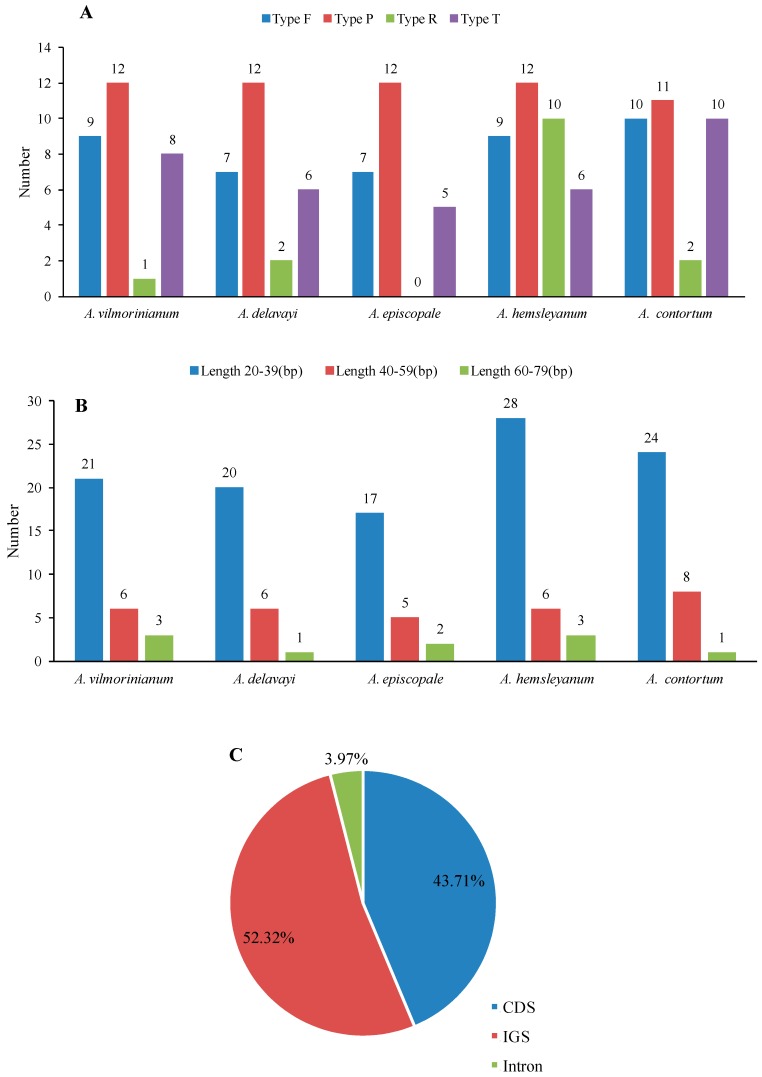
The type, length and distribution of repeats in the chloroplast genomes of five *Aconitum* species. (**A**) Number of different repeat types: F, forward; P, palindromic; R, reverse; T, tandem; (**B**) Number of different repeat lengths; (**C**) Proportion of repeats in LSC, SSC and IR regions.

Repeat regions are considered to play an important role in the generation of substitutions and indels [29,31,32]. A total of 151 repeats were identified in the five *Aconitum* chloroplast genomes, including 42 forward repeats, 59 palindromic repeats, 15 reverse repeats and 35 tandem repeats. *Aconitum hemsleyanum* possessed the highest number of repeats (37), while *A. episcopale* possessed the fewest (24) (Figure 3A and Table S3). The majority of repeats ranged in size from 20 to 39 bp (Figure 3B). Most of them were distributed in intergenic (52.32%) or gene regions (43.71%) and only 3.97% were located in intron regions, such as *clpP* and *rpoC1* (Figure 3C and Table S3). Repeats located in identical regions with the same lengths were identified as shared repeats. Using this criterion, 18 repeats were found to be shared by all five *Aconitum* species (Table 3), providing a useful resource for phylogeny and population studies.

**Figure 4:**
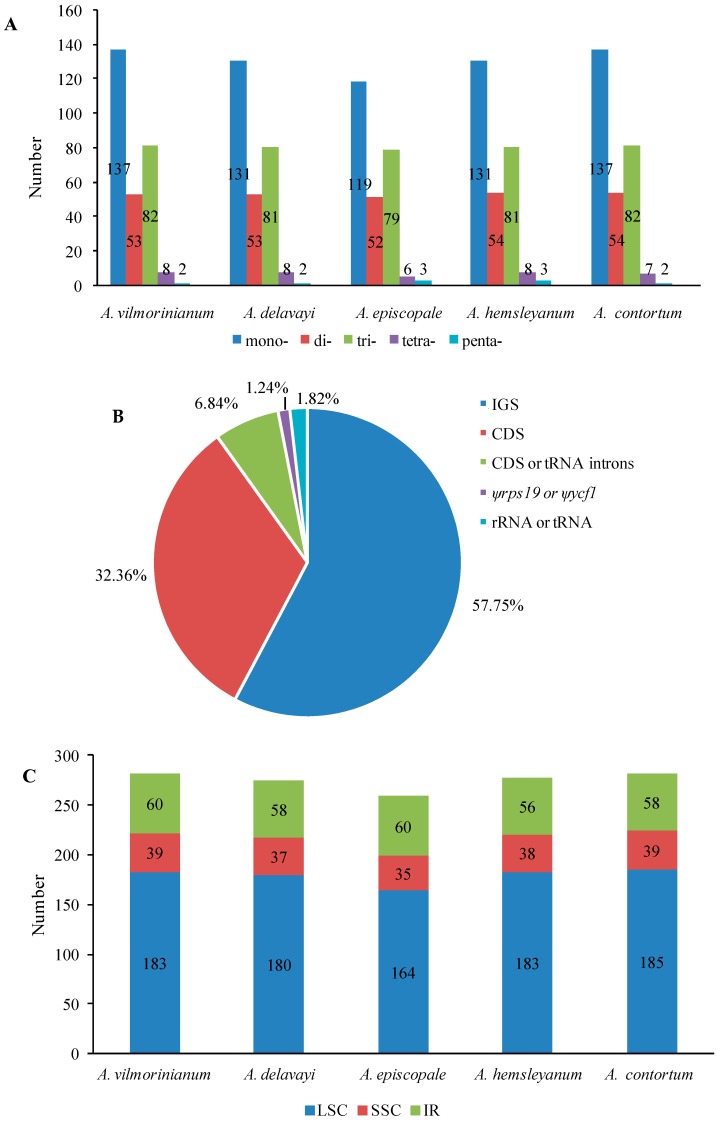
The number and distribution of SSRs in the chloroplast genomes of five *Aconitum* species. (**A**) Total number of repeats; (**B**) Proportion of repeats in IGS, CDS, CDS or tRNA introns, *ψrps19* or *ψycf1* and rRNA or tRNA regions; (**C**) Number of repeats in LSC, SSC and IR.

SSRs are tandemly repeated DNA sequences with 1–6 bp and are distributed throughout the genome. They are widely used for the screening of effective molecular markers for detecting intraspecific and interspecific polymorphisms [33,34] and population genetics [35]. In total, 1375 SSRs were identified in the cp genome of the five *Aconitum* species, ranging from 259 SSRs in *A. episcopale* to 282 SSRs in *A. vilmorinianum* and *A. contortum*; more than half of the SSRs were composed of A or T (Figure 4A,C and Table S4). The majority of SSRs were mononucleotide repeats, followed by trinucleotide repeats; no hexanucleotide repeats were found. Most of the SSR repeats were located in intergenic spacer regions (IGS) (57.75%), while the regions situated in coding DNA sequence (CDS) or tRNA introns, *ψrps19* or *ψycf1* and rRNA or tRNA accounted for 32.36%, 6.84%, 1.24% and 1.82% of SSR repeats, respectively (Figure 4B). Among these mononucleotide repeats, there were generally polyadenine (polyA, 47.33–51.09%) and polythymine (polyT, 45.99–49.58%) repeats (Table S4) and rarely tandem guanine (G) or cytosine (C) repeats, which agrees with previous chloroplast SSRs reports [36]. The longest polyA (17 bp) and the most abundant mononucleotide (51.09%) were found in *A*. *contortum*.

### 2.4. Comparative Chloroplast Genomic Analysis

**Figure 5:**
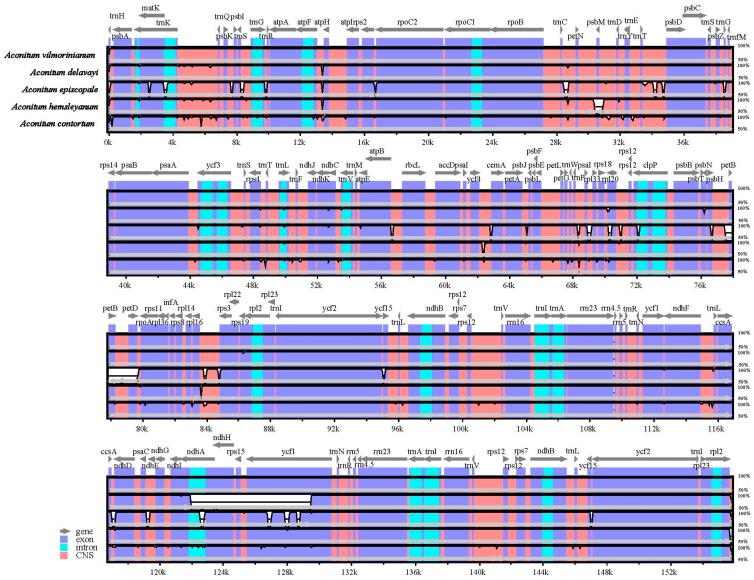
Comparison of five chloroplast genomes using *A. vilmorinianum* annotation as a reference. The vertical scale indicates the percentage of identity, ranging from 50 to 100%. The horizontal axis indicates the coordinates within the chloroplast genome. Genome regions are color-coded as exons, introns and conserved non-coding sequences (CNS).

**Figure 6:**
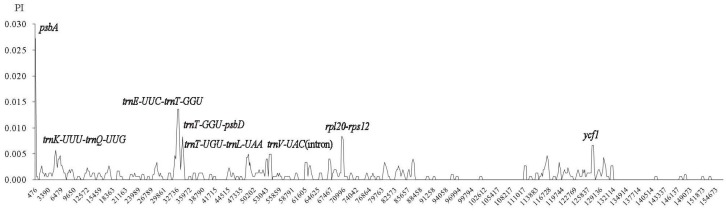
Sliding-window analysis on the cp genomes for five *Aconitum* species. X-axis: position of the midpoint of a window; Y-axis: nucleotide diversity (Pi) of each window.

Sequence identity plots of the five *Aconitum* species were generated, with the annotation of *A. vilmorinianum* cp genome as a reference (Figure 5). LSC and SSC regions were more divergent than IRs regions. Whereas, the coding regions were more conserved than the non-coding regions, the highly divergent non-coding regions among the five chloroplast genomes appeared in IGS, such as *trnH*-*psbA*, *trnK*-*trnQ*, *atpF*-*atpH*, *trnC*-*petN*, *ycf4*-*cemA*, *trnP*-*psaJ* and *rpl16*-*rps3*. Among coding regions, *ndhA*, *ndhH*, *rps15* and *ycf1* genes were relatively divergent. On the other hand, all the rRNA genes were highly conserved and are similar to other plants’ chloroplast genomes [37]. For further understanding of the nucleotide variability (Pi), we also calculated the DNA polymorphism among these five *Aconitum* species (Figure 6). The results are the same as previous reports: the IR regions more conserved than LSC and SSC regions [13,14]. There were eight variable regions that showed high Pi values (≥0.005), including *psbA* and *ycf1* genes, the intron of *trnV-UAC* and intergenic regions (*trnK-UUU*-*trnQ-UUG*, *trnE-UUC*-*trnT-GGU*, *trnT-GGU*-*psbD*, *trnT-UGU*-*trnL-UAA* and *rpl20*-*rps12*) in the chloroplast genomes. These hotspot regions could be developed as molecular markers and barcoding for future phylogenetic analyses and species identification of *Aconitum*.

### 2.5. IR Expansion and Contraction

**Figure 7:**
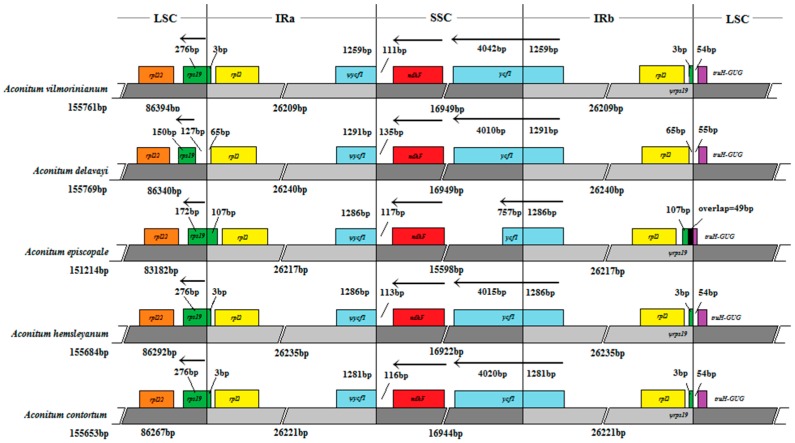
Comparisons of LSC, SSC and IR region borders among five *Aconitum* chloroplast genomes. ψ indicates a pseudogene. Genes are denoted by colored boxes. The number above the gene features shows the distance between the end of the gene and the borders sites. The slashes indicate the location of the distance. The arrows indicated the orientation (5’→3’) of the *rps19*, *ndhF* and *ycf1* genes. This figure is not to scale.

IRs are the most conserved regions of the chloroplast genome. However, the contraction and expansion of IR borders are common evolutionary events and are the major reason for size differences between chloroplast genomes [37]. Chloroplast genome structure and the junction positions between IR regions were well conserved among the five *Aconitum* species but structure variation was still found in the IRs/SC borders (Figure 7). There were 3 bp protrusion of rps19 gene into IRa regions, with the corresponding pseudogene fragment *ψrps19*, located in the IRa/LSC border for *A. vilmorinianum*, *A. hemsleyanum* and *A. contortum*. This agrees with most *Aconitum* subgen. *Aconitum* species. However, the length of this protrusion was 107 bp in *A. episcopale* [13]. Moreover, no pseudogene fragment *ψrps19* was found in *A. delavayi* and there were 127 bp between *rps19* and the IRa/LSC border. Long *ψycf1* fragments with 1259–1291 bp were created at the IRa regions due to the border between SSC and IRb located in the *ycf1* genes. In addition, the *trnH-GUG* genes for four of the five species were all located in the LSC region, with the distance between *trnH-GUG* and the IRb/LSC border varying from 54 to 55 bp. The exception to this was *A. episcopale*, in which the *trnH-GUG* gene overlapped with the *ψrps19* by 49 bp.

### 2.6. Phylogenetic Analyses

**Figure 8:**
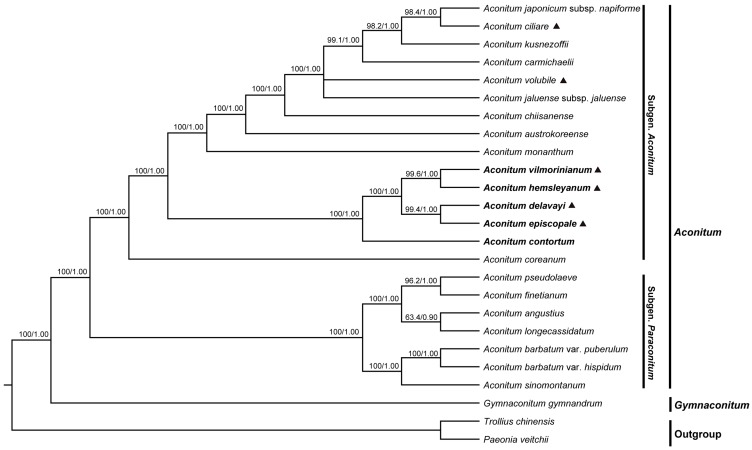
Phylogenetic tree constructed using Maximum Likelihood (ML) and Bayesian Inference (BI) methods, based on the whole cp genomes from different species. The numbers above the branches represent ML bootstrap values/BI posterior probabilities. Triangle: twining species belonging to subgen. *Aconitum.*

Relationships within *Aconitum* species are fairly well resolved in previously published studies but the positions of the twining species of *Aconitum* still remain somewhat uncertain and there is a possibility that they might have evolved independently in various groups [13,23]. The phylogenetic analysis of these five species mostly used the chloroplast fragment *trnH-psbA* and the nuclear fragment ITS [21,23,24]. In the present study, we chose two datasets (the whole cp genomes and 77–79 PCGs) from the five *Aconitum* species and 20 published plastomes to perform the phylogenetic analysis. A phylogenetic tree based on the same dataset, using ML and BI, had an almost identical topological structure but different support values (Figure 8). 

There were no obvious conflicts between the phylogenetic trees built by different datasets but the support values of the branches based on the whole cp genomes dataset were higher than those based on the PCG dataset, except for the clade of *A*. *delavayi* and *A*. *episcopale*. All of the 22 *Aconitum* taxa formed a monophyletic clade with 100% bootstrap value or the Bayesian posterior probability. The five species formed a monophyletic clade with 100% bootstrap value or the Bayesian posterior probability within *Aconitum* subgen. *Aconitum*, among which three twining species belonging to ser. *Volubilia* (*A. episcopale*, *A*. *vilmorinianum* and *A*. *hemsleyanum)* were clustered with the species from ser. *Stylosa* (*A*. *contortum*) and ser. *Ambigua* (*A*. *delavayi*). Furthermore, *A. delavayi* and *A. episcopale* formed a monophyletic group with very high support value and sister to the clade that was clustered by *A*. *vilmorinianum* and *A*. *hemsleyanum* (Figure 8), a finding consistent with the previous result that the ser. *Volubilia* was not a monophyly [23]. *Aconitum vilmorinianum* had a closer phylogenetic relationship to *A*. *hemsleyanum* than to the other three species. At the same time, the phylogenetic relationship constructed using the whole cp genomes dataset showed that *A. ciliare* and *A. japonicum* subsp. *napiforme* formed a clade with a high support value of 98.4/1.00, sister to *A. kusnezoffii* (Figure 8); however, their monopyly was not resolved with the PCGs (Figure S1). The current phylogenetic tree showed the deep-level relationships of *Aconitum* species, raising the possibility that the cp genome sequences may be useful for elucidating the phylogeny of *Aconitum* species in the future.

## 3. Materials and Methods

### 3.1. Plant Material, DNA Extraction and Sequencing 

Fresh leaves of five *Aconitum* species were collected from Yunnan province and dried with silica gel. Voucher specimens were deposited in the herbarium of the Kunming Institute of Botany (KUN), Chinese Academy of Sciences. Total genomic DNA was extracted with the modified cetyltrimethyl ammonium bromide (CTAB) method [38]. The extracted DNA was sequenced using the Illumina Miseq platform (Illumina, San Diego, CA, USA). The chloroplast sequence generated in this study was submitted to GenBank (Table 1).

### 3.2. Chloroplast Genome Assembly and Annotation

For each *Aconitum* species, reads of the cp genome were assembled using CLC Genomic Workbench v10 (CLC Bio., Aarhus, Denmark). All the contigs were checked against the reference genome of *A. chiisanense* (KT820665), using BLAST (https://blast.ncbi.nlm.nih.gov/) and aligned contigs were oriented according to the reference genome. The complete cp genomes were then constructed using Geneious v4.8.5 (Biomatters Ltd., Auckland, New Zealand). 

The annotation of cp genome sequence was performed using DOGMA (http://dogma.ccbb.utexas.edu/) [39] and start/stop codons and intron/exon boundaries were adjusted in Geneious v4.8.5. The tRNA was identified through tRNAscan-SE v2.0 [40]. The circular genome map was generated in OGDRAW (http://ogdraw.mpimp-golm.mpg.de/) [41]. 

### 3.3. Structure of Genome and Genome Comparison 

All protein-coding genes were used for determining the codon usage. Avoiding the influence of the amino acid composition, we examined the RSCU using MEGA v7.0 [42]. We identified the repeat sequences, including palindromic, reverse and forward repeats, in REPuter Online software, with the following settings: Hamming distance of 3 and minimum repeat size of 30 bp [43]. Tandem Repeats Finder v4.07 was used to analyze tandem repeats using default settings [44]. Simple sequence repeats (SSRs) were detected by Phobos v3.3.12 [45] and SSR Hunter v1.3 [46]. The threshold value of the repeat number was set as: ≥8 for mononucleotide repeats, ≥4 for dinucleotide repeats and ≥3 for trinucleotide repeats, tetranucleotide repeats, pentanucleotide repeats and hexanucleotide repeats. The mVISTA was used to compare the cp genomes of the five *Aconitum* species in Shuffle-LAGAN mode, with annotation of *A. vilmorinianum* as a reference [47]. These cp genome sequences were aligned by CLC Genomic Workbench v10. Sliding window analysis was conducted to determine the nucleotide diversity of the cp genome using DnaSP v5, with 200 bp of step size and 600 bp window length [48].

### 3.4. Phylogenetic Analyses

In order to explore the phylogenic relationships of the five species among *Aconitum*, a total of 20 complete cp genomes of the family Ranunculaceae were obtained from GenBank, including 17 *Aconitum* taxa and another three species from different genera as outgroups (Table S5). For the phylogenetic analysis, 77–79 PCGs and the whole cp genomes were aligned by CLC Genomic Workbench with default parameters. A Maximum Likelihood (ML) tree was then performed in RAxML [49], with the nucleotide substitution model of GTR + Gamma and a bootstrap of 1000 replicates. Bayesian inference (BI) was estimated with MrBayes v3.2.6 [50]. The best-fitting substitution model was selected using jModelTest [51]. The general time-reversible (GTR) model was chosen with a gamma model for the rate of heterogeneity. The Markov chain Monte Carlo (MCMC) analysis was run for 10,000,000 generations. The trees were sampled every 1000 generations, with the first 25% discarded as burn-in.

## 4. Conclusions

The cp genomes of the four special *Aconitum* medicinal species ‘Dula’ and one species *A*. *hemsleyanum* were reported for the first time. The cp genomes all displayed a typical quadripartite structure, which was similar to that of most angiosperms. *Aconitum episcopale* was found to have the smallest size of cp genome (151,214 bp) presently known in *Aconitum* species. Except for *A. delavayi*, the other four species were found to have two pseudogenes (*ψrps19* and *ψycf1*). Eight variable regions (*psbA*, *ycf1*, *trnV*, *trnK*-*trnQ*, *trnE*-*trnT*, *trnT*-*psbD*, *trnT*-*trnL* and *rpl20*-*rps12*) were identified and may potentially be useful as specific DNA barcodes for identifying *Aconitum* species. The result of phylogenetic analyses showed that the *Aconitum* subgen. *Aconitum* ser. *Volubilia* was not monophyletic. The resulting trees showed good construction of the deep-level relationships of *Aconitum* species, indicating that the whole cp genome sequences will have much better resolution in the phylogenetic study of *Aconitum* species in the future.

## Figures and Tables

**Table 1:** The basic characteristics of chloroplast genomes of five *Aconitum* species.

Characteristics	*A. vil*	*A. del*	*A. epi*	*A. hem*	*A. con*
Location	Wuding	Heqing	Dali	Qiaojia	Dali
Voucher specimens	LCF1	1395	1379	QJ6	895
GenBank numbers	MG678799	MG678802	MG678801	MG678800	MG678803
Total clean reads	8,783,602	24,899,740	22,334,862	14,157,482	19,869,478
Number of contigs	2540	1468	1190	1161	1749
Contigs used for constructing the cp genome	4	3	3	3	3
N50 of contigs (bp)	1612	369	1939	1966	1953
Cp genome coverage (×)	56.4	159.9	147.7	90.9	127.7
Total cp DNA size (bp)	155,761	155,769	151,214	155,684	155,653
LSC size (bp)	86,394	86,340	83,182	86,292	86,267
IR size (bp)	26,209	26,240	26,217	26,235	26,221
SSC size (bp)	16,949	16,949	15,598	16,922	16,944
Total number of genes	132	131	131	132	132
Number of different protein-coding genes	78	78	78	78	78
Number of different tRNA genes	30	30	29	30	30
Number of different rRNA genes	4	4	4	4	4
Number of duplicated genes	20	19	20	20	20
Total number of pseudogenes	2	1	2	2	2
GC content (%)	38.1	38.1	38.3	38.1	38.1
GC content of LSC (%)	36.2	36.2	36.4	36.2	36.2
GC content of IR (%)	43.0	43.0	42.9	43.0	43.0
GC content of SSC (%)	32.5	32.6	32.9	32.6	32.6

cpDNA: chloroplast genome DNA; LSC: large single copy; IR: inverted repeat; SSC: small single copy. *A. vil*: *A. vilmorinianum* Komarov, *A. del*: *A. delavayi* Franchet, *A. epi*: *A. episcopale* H. Léveillé, *A. hem*: *A. hemsleyanum* E. Pritzel, *A. con*: *A. contortum* Finet & Gagnepain.

**Table 2:** A list of genes found in the chloroplast genomes of five *Aconitum* species.

Category	Grope of Genes	Name of Genes
Transcription and translation	Ribosomal proteins (LSU)	rpl2*(×2), *rpl14*, *rpl16*, *rpl20*, *rpl22*, *rpl23*(×2), *rpl33*, *rpl36*
	Ribosomal proteins (SSU)	*rps2*, *rps3*, *rps4*, *rps7*(×2), *rps8*, *rps11*, rps12**(×2), *rps14*, *rps15*, *rps18*, *ψrps19*
	RNA polymerase	*rpoA*, *rpoB*, rpoC1*, *rpoC2*
	Translational initiation factor	*infA*
	rRNA genes	*rrn16*(×2), *rrn23*(×2), *rrn4.5*(×2), *rrn5*(×2)
	tRNA genes	trnA-UGC*(×2), *trnC-GCA*, *trnD-GUC*, *trnE-UUC*, *trnF-GAA*, *trnfM-CAU*, trnG-GCC*, *trnG-UCC*, *trnH-GUG*, *trnI-CAU*(×2), trnI-GAU*(×2), trnK-UUU*, *trnL-CAA*(×2), trnL-UAA*, *trnL-UAG*, *trnM-CAU*, *trnN-GUU*(×2), *trnP-UGG*, *trnQ-UUG*, *trnR-ACG*(×2), *trnR-UCU*, *trnS-GCU*, *trnS-GGA*, *trnS-UGA*, *trnT-GGU*, *trnT-UGU*, *trnV-GAC*(×2), trnV-UAC*, *trnW-CCA*, *trnY-GUA*
Photosynthesis	Photosystem I	*psaA*, *psaB*, *psaC*, *psaI*, *psaJ*
	Photosystem II	*psbA*, *psbB*, *psbC*, *psbD*, *psbE*, *psbF*, *psbH*, *psbI*, *psbJ*, *psbK*, *psbL*, *psbM*, *psbN*, *psbT*, *psbZ*
	NADH oxidoreductase	ndhA*, ndhB*(×2), *ndhC*, *ndhD*, *ndhE*, *ndhF*, *ndhG*, *ndhH*, *ndhI*, *ndhJ*, *ndhK*
	Cytochrome b6/f complex	petA*, petB*, *petD*, *petG*, *petL*, *petN*
	ATP synthase	*atpA*, *atpB*, *atpE*, atpF*, *atpH*, *atpI*
	Rubisco large subunit	*rbcL*
	ATP-dependent protease subunit gene	clpP**
Other genes	Maturase	*matK*
	Envelop membrane protein	*cemA*
	Subunit Acetyl- CoA-Carboxylate	*accD*
	c-type cytochrome synthesis gene	*ccsA*
Unknown	Conserved Open reading frames	*ψycf1*, *ycf2*(×2), ycf3**, *ycf4*, *ycf15*(×2)

* contains one intron, ** contains two introns, (×2) shows genes duplicated in the IR regions, ψ shows pseudogenes, *A*. *delavayi* did not contain *ψrps19*, *A*. *episcopale* not contanin *trnG-UCC*.

**Table 3:** The shared repeats of five *Aconitum* species.

No.	Size (bp)	Units	Type	Location Region
1	30	TAAAC(A)GGAA(G)AGAGAGGGATTCGAACCCTCG	F	IGS(*psbI*,*trnS-GCU*), IGS(*psbC*,*trnS-UGA*)
2	52	AGAAAAAGAATTGCAATAGCTAAATGG(A)TGA(G)TGA(C)GCAATATCGGTCAGCCATA	F	*psaB*(CDS),*psaA*(CDS)
3	39	CAGAACCGTACATGAGATTTTCACCTCATACGGCTCCTC	F	*ycf3*(intron), IGS(*rps12*,*trnV-GAC*)
4	31	CC(G)ATATTGATGATAGTGAC(G)GATATT(C)GATGA	F	*ycf2*(CDS)
5	42	TGGTTGTTCGCCGTTCAAGAATTCTTGAACGGCGAACAACCA	F	*ycf15*(CDS)
6	31	ATCATCG(A)ATATCC(G)TCACTATCATCAATATC(G)G	F	*ycf2*(CDS)
7	32	GAGATTTTATTTCG(A)AATTTGAAATAAAATCTC	P	IGS(*psbI*,*trnS-GCU*)
8	30	ACGGAAAGAGAGGGATTCGAACCCTCGGTA	P	IGS(*psbI*,*trnS-GCU*), IGS(*trnS-GGA*,*rps4*)
9	30	AA(C)GGAG(A)AGAGAGGGATTCGAACCCTCGA(G)TA	P	IGS(*trnSUGA*,*psbZ*), IGS(*trnS-GGA*,*rps4*)
10	39	CAGAACCGTACATGAGATTTTCACCTCATACGGCTCCTC	P	*ycf3*(intron), IGS(*trnV-GAC*,*rps12*)
11	72	GTAAGAATAAGAACTCAATGGACCTTGCCCCTCG(A)AATTT(C)GAGGGGCAAGGTCCATTGAGTTCTTATTCTTAC	P	IGS(*petA*,*psbJ*)
12	48	ATGTATCTAGGGACTAGTCGCTTC(G)C(G)AAGCGACTAGTCCCTAGATACAT	P	IGS(*petD*,*rpoA*)
13	31	CCATATTGATGATAGTGACGATATTGATGAT	P	*ycf2*(CDS)
14	31	CGATATTGATGATAGTGAGGATATCGATGAT	P	*ycf2*(CDS)
15	42	TGGTTGTTCGCCGTTCAAGAATTCTTGAACGGCGAACAACCA	P	*ycf15*(CDS)
16	42	TGGTTGTTCGCCGTTCAAGAATTCTTGAACGGCGAACAACCA	P	*ycf15*(CDS)
17	38	TACACATGAAGTAAAGAAA×2	T	IGS(*trnS-GCU*,*trnG-GCC*)
18	26	TTTTATAGTTAAA×2	T	*clpP*(intron)

IGS: intergenic spacer regions; CDS: coding DNA sequence.

## Supplementary Materials

Supplementary materials will be available online.
