# Supplementary figures and images for: Comparative Analysis of the Complete Chloroplast Genomes of Four Aconitum Medicinal Species
Source: Molecules. 2018 Apr 26;23(5):1015. doi: 10.3390/molecules23051015 (PMC6102581; doi:10.3390/molecules23051015)

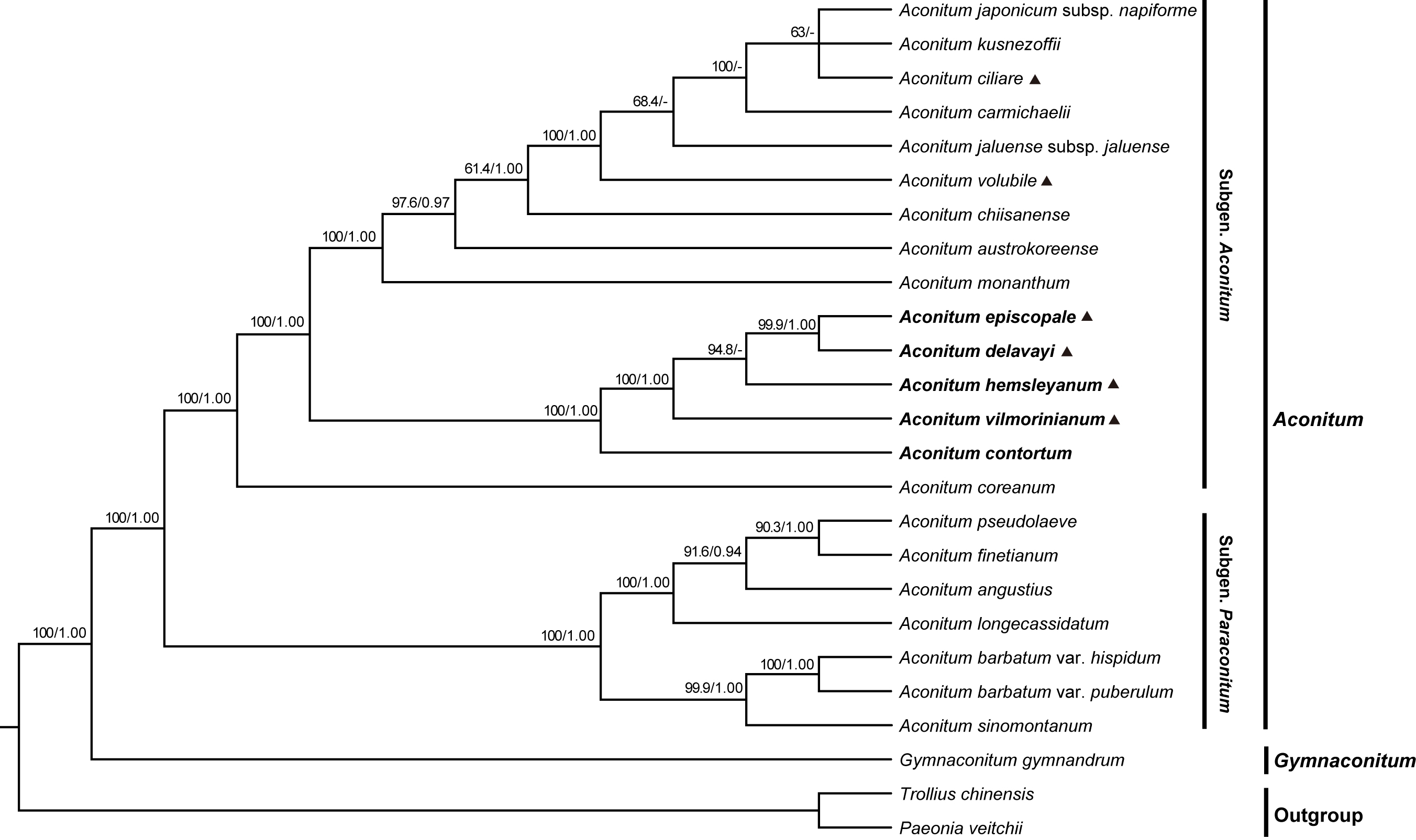

Supplement: Supplementary file 1 [file molecules-23-01015-s001.zip › Supplementary revision/Figure S1 Phylogenetic tree constructed using Maximum Likelihood (ML) and Bayesian Inference (BI) methods, based on the PCGs sequences from different species.jpg]
