# Supplementary material for: Comparative Analysis of the Complete Chloroplast Genomes of Four Aconitum Medicinal Species
Source: Molecules. 2018 Apr 26;23(5):1015. doi: 10.3390/molecules23051015 (PMC6102581; doi:10.3390/molecules23051015)
Supplement: Supplementary file 1 [file molecules-23-01015-s001.zip › Supplementary revision/Table S5 GenBank accession numbers of the chloroplast genomes used in this study.doc]

Table S5 GenBank accession numbers of the chloroplast genomes used in this study

| Plant | Genbank Accession | Length |
| --- | --- | --- |
| *Aconitum chiisanense* | KT820665 | 155,934 bp |
| *Aconitum carmichaelii* | [KX347251](https://www.ncbi.nlm.nih.gov/nuccore/KX347251) | 155,737 bp |
| *Aconitum austrokoreense* | [KT820663](https://www.ncbi.nlm.nih.gov/nuccore/KT820663). | 155,682bp |
| *Aconitum ciliare* | [KT820666](https://www.ncbi.nlm.nih.gov/nuccore/KT820666) | 155,832 bp |
| *Aconitum coreanum* | [KT820667](https://www.ncbi.nlm.nih.gov/nuccore/KT820667) | 157,029 bp |
| *Aconitum kusnezoffii* | [KT820671](https://www.ncbi.nlm.nih.gov/nuccore/KT820671) | 155,862 bp |
| *Aconitum monanthum* | [KT820672](https://www.ncbi.nlm.nih.gov/nuccore/KT820672) | 155,688 bp |
| *Aconitum* *longecassidatum* | [KY407561](https://www.ncbi.nlm.nih.gov/nuccore/KY407561) | 155,524 bp |
| *Aconitum* *pseudolaeve* | [KY407562](https://www.ncbi.nlm.nih.gov/nuccore/KY407562) | 155,628 bp |
| *Aconitum barbatum* var. *puberulum* | KC_844054 | 156,749 bp |
| *Aconitum barbatum* var. *hispidum* | KT_820664 | 156,782 bp |
| *Aconitum jaluense* subsp. *jaluense* | KT_820669 | 155,926 bp |
| *Aconitum japonicum* subsp. *napiforme* | KT_820670 | 155,878 bp |
| *Aconitum* *volubile* | KU_556690 | 155,872 bp |
| *Aconitum angustius* | MF_155664 | 156,109 bp |
| *Aconitum finetianum* | MF_155665 | 155,625 bp |
| *Aconitum sinomontanum* | MF_155666 | 157,215 bp |
| *Gymnaconitum gymnandrum* | [KT964697](https://www.ncbi.nlm.nih.gov/nuccore/KT964697) | 157,327 bp |
| *Paeonia veitchii* | [KT894821](https://www.ncbi.nlm.nih.gov/nuccore/KT894821) | 152,682 bp |
| *Trollius chinensis* | [KX752098](https://www.ncbi.nlm.nih.gov/nuccore/KX752098) | 160,191 bp |
